# Ketone Monoester Supplementation Does Not Expedite the Recovery of Indices of Muscle Damage After Eccentric Exercise

**DOI:** 10.3389/fnut.2020.607299

**Published:** 2020-12-08

**Authors:** Patrick W. Martin-Arrowsmith, Jamie Lov, Jiaying Dai, José A. Morais, Tyler A. Churchward-Venne

**Affiliations:** ^1^Department of Kinesiology and Physical Education, McGill University, Montreal, QC, Canada; ^2^Division of Geriatric Medicine, McGill University, Montreal, QC, Canada; ^3^Research Institute of the McGill University Health Centre, Montreal, QC, Canada

**Keywords:** ketone supplements, ketosis, post-exercise recovery, muscle damage, eccentric exercise, exercise performance

## Abstract

**Purpose:** The purpose of this study was to evaluate the effects of a ketone monoester supplement on indices of muscle damage during recovery after eccentric exercise.

**Methods:** In a randomized, double-blind, independent group design, 20 moderately active healthy young adults consumed 360 mg per kg^−1^ bodyweight of a ketone monoester (KET) or energy-matched carbohydrate (CON) supplement twice daily following eccentric exercise (drop jumps). Maximal isometric voluntary contraction (MIVC) torque, counter-movement jump (CMJ) height, and muscle soreness were measured before (PRE), and immediately (POST), 24 h and 48 h post-exercise. Blood samples were collected for analysis of β-hydroxybutyrate (β-OHB), creatine kinase (CK), and select pro- and anti-inflammatory cytokines.

**Results:** Peak blood β-OHB concentration after supplement intake was greater (*P* < 0.001) in KET (4.4 ± 0.8 mM) vs. CON (0.4 ± 0.3 mM). Exercise increased CK concentration at 24 h and 48 h vs. PRE (time: *P* < 0.001) with no difference between KET and CON. Exercise reduced MIVC (KET: −19.9 ± 14.6; CON: −22.6 ± 11.1%) and CMJ (KET: −11.0 ± 7.5; CON: −13.0 ± 8.7%) at POST relative PRE; however, there was no difference between KET and CON on the recovery of MIVC at 24 h (KET: −15.4 ± 20.4; CON: −18.7 ± 20.1%) or 48 h (KET: −7.2 ± 21.2; CON: −11.8 ± 20.2%), or CMJ at 24 h (KET: −9.2 ± 11.5; CON: −13.4 ± 10.8) or 48 h (KET: −12.5 ± 12.4; CON: −9.1 ± 11.7). Muscle soreness was increased during post-exercise recovery (time: *P* < 0.001) with no differences between KET and CON. Monocyte chemoattractant protein-1 was greater (group: *P* = 0.007) in CON (236 ± 11 pg/mL) vs. KET (187 ± 11 pg/mL).

**Conclusion:** In conclusion, twice daily ingestion of a ketone monoester supplement that acutely elevates blood β-OHB concentration does not enhance the recovery of muscle performance or reduce muscle soreness following eccentric exercise in moderately active, healthy young adults.

## Introduction

Exercise-induced muscle damage (EIMD) is a non-pathological condition that occurs in response to unaccustomed exercise involving repeated eccentric (i.e., lengthening) muscle contractions. The main consequences of EIMD are a temporary reduction in muscle function (i.e., force production) and increased muscle soreness accompanied by an increase in intramuscular proteins [e.g., creatine kinase (CK)] in the blood and swelling of the involved muscle group (1). Consequently, EIMD may negatively impact the ability to perform during subsequent training/exercise sessions. The mechanisms of EIMD are complex but are thought to involve both a primary and secondary phase of damage (2, 3). The primary phase of EIMD involves ultrastructural damage to the muscle and/or impairments in excitation-contraction coupling due to the mechanical work performed during exercise (2, 3). The secondary phase of EIMD occurs in response to the primary phase and involves biochemical changes, the hallmarks of which are inflammation and oxidative stress, that may further exacerbate damage to the muscle and surrounding tissue (4). Given the negative symptoms associated with EIMD, there is significant interest among athletes, coaches, and the broader fitness community on novel therapeutic approaches that may effectively reduce indices of EIMD and accelerate recovery after eccentric exercise.

A variety of nutritional interventions have been evaluated in an attempt to alleviate muscle soreness and impairments in muscle function during recovery from eccentric exercise [for reviews see (2, 5–7)]. From an EIMD perspective, nutritional interventions that increase muscle protein synthesis and/or reduce protein breakdown may support recovery from damage by increasing the rate of tissue repair/remodeling or reducing the extent of damage (8). In addition, interventions that can reduce EIMD-induced increases in inflammation and/or oxidative stress may minimize further exacerbation of secondary muscle damage, and thereby contribute to improved short-term recovery (2, 9). Ketone bodies [i.e., D-β-hydroxybutyrate (β-OHB), acetoacetate, and acetone] are lipid-derived molecules that are normally produced in the body in response to starvation or carbohydrate restriction (i.e., a ketogenic diet). Under these conditions they serve as an important fuel source for metabolically active tissues including the brain and skeletal muscle. Recently, exogenous ketone “supplements” have been developed, including the ketone monoester (R)-3-hydroxybutyl (R)-3-hydroxybutyrate (10), that rapidly increases β-OHB to ~3–5 mM within ~30 min in healthy humans (11) without the need for dietary restriction. While mostly studied in terms of their acute effects on endurance exercise performance (12, 13), exogenous ketone supplements and increases in ketone body availability have been proposed to play a role in modulating recovery after exercise (14, 15). Ketone bodies are increasingly understood to act as important signaling molecules that can regulate protein metabolism, inflammation, and oxidative stress (16); putative factors involved in recovery following EIMD. For example, β-OHB has been shown to stimulate skeletal muscle protein synthesis in humans (17), and reduce muscle (18), and whole-body protein breakdown (18, 19). Anticatabolic effects of β-OHB have also recently been demonstrated in human skeletal muscle under inflammatory insult (18). Ketone bodies have been reported to mitigate inflammation and oxidative stress both *in-vitro* and *in-vivo* (20) and may stimulate muscle regeneration or remodeling by enhancing satellite cell activation and differentiation (21, 22). Collectively, these actions indicate a potential role for exogenous ketone supplementation and elevated β-OHB as a novel therapeutic approach to expedite the recovery of indices of EIMD after eccentric exercise.

The purpose of this study was to evaluate the effects of supplementation with a ketone monoester, that acutely increases blood β-OHB concentration, on indices of EIMD including muscle dysfunction and muscle soreness during recovery from a bout of eccentric exercise in healthy young moderately-active adults. We hypothesized that twice daily supplementation with a ketone monoester (KET) would reduce the concentration of select pro-inflammatory cytokines, expedite the recovery of muscle function, and reduce muscle soreness when compared to supplementation with an energy-matched carbohydrate supplement (CON).

## Materials and Methods

### Participants

Twenty healthy young men and women (*n* = 10 men and 10 women) 18–35 years of age, with a BMI >18.5 and <30.0 kg/m^2^ participated in this randomized, double-blind, parallel group study. Participants were moderately active (defined as regularly exercising 2–4 times per week) but unaccustomed to high-force plyometric exercise. Participants were excluded if they met any of the following criteria: identified metabolic or intestinal disorder, use of tobacco products, adherence to a ketogenic, vegetarian, or vegan diet, use of certain medications (i.e., corticosteroids, non-steroidal anti-inflammatories, or prescription strength acne medications), musculoskeletal ailments that prevented them from performing the required exercise, pregnancy, asthma, and use of certain dietary supplements (i.e., creatine, fish oil, beta-alanine). Female participants were studied during the early follicular phase of their menstrual cycle (within 5 days of the onset of menses). Participants' characteristics are presented in Table 1. All participants were informed about the purpose of the study, the experimental procedures, and possible risks prior to providing informed written consent to participate.

**Table 1:** Participant characteristics and baseline test scores.

	**KET (*n* = 10)**	**CON (*n* = 10)**	***P***
Age, years	25, 5	24, 4	0.423
Body mass, kg	72.6, 12.8	70.7, 11.3	0.727
Height, cm	169, 10	172, 7	0.391
BMI, kg/m^2^	24.3, 2.2	23.3, 2.3	0.319
Body fat, %	27.3, 8.1	26.1, 7.2	0.738
Lean mass, kg	49.5, 11.1	49.4, 9.2	0.975
Heart rate, bpm	65, 15	68, 10	0.683
Systolic BP, mmHg	113, 7	113, 7	0.924
Diastolic BP, mmHg	71, 9	70, 8	0.770
MIVC, Nm	205.9, 61.6	229.9, 64.1	0.405
CMJ, cm	31.0, 8.6	33.5, 9.6	0.497
VAS, mm	10, 7	9, 7	0.572
PPT, kPa	531, 276	333, 121	0.053
Thigh circumference, cm	56.7, 4.4	55.6, 4.4	0.582
Calf circumference, cm	38.4, 2.2	38.2, 2.2	0.804
Flexibility, degrees	125, 10	125, 9	0.907
BAM+, AU	43.7, 20.8	49.4, 15.4	0.489

*Values are mean ± SD. Data were analyzed with an unpaired t-test. AU, arbitrary unit; BAM+, brief assessment of mood adapted; CMJ, counter-movement jump; CON, carbohydrate control group; KET, ketone monoester group; MIVC, maximal isometric voluntary contraction; PPT, pressure-pain threshold; VAS, visual analog scale*.

### Research Ethics Approval

The study was approved by the Faculty of Medicine Institutional Review Board at McGill University on January 14, 2019 (IRB Study Number: A01-M01-19A). All participants provided written informed consent prior to study participation. The study was conducted in accordance with the ethical standards of the Faculty of Medicine Institutional Review Board at McGill University on human experimentation and in accordance with the Helsinki Declaration of 1975 as revised in October 2013.

### Preliminary Testing

Participants underwent an initial screening and familiarization visit during which height (via a wall-mounted stadiometer), weight (via a digital balance), heart rate and blood pressure (Omron 10 series, Model BP786CANN), and body composition (by dual-energy X-ray absorptiometry; GE Healthcare, Madison, WI, USA) were assessed. Participants were also thoroughly familiarized with the exercise testing equipment and procedures. Participants received a demonstration by one of the study investigators and then performed a guided familiarization trial for the performance measures. Participants were deemed healthy based on their responses to a medical questionnaire and screening results. The initial screening and familiarization visit, and onset of the experiment were separated by at least 4 days.

### Diet and Physical Activity

Study participants were asked to refrain from strenuous physical activity and alcohol consumption for 2 days immediately prior to the onset of the experiment and during all experimental test days. In addition, participants were required to complete food intake and physical activity questionnaires during this time. Average dietary intake prior to (pre-study phase) and during (EIMD recovery phase) the experiment was analyzed using commercially available software (Food Processor version 11.7; ESHA Research; Salem OR, USA). On the evening before each experimental test day, participants were instructed to stop consuming food or beverages other than water or their nutritional treatment (described below) by 20:00 h, after which they remained fasted until testing the following morning.

### Overview of Study Design

The present study utilized a randomized, double-blind, independent group design. One group (KET) received a ketone monoester, while the other group (CON) received an energy-matched amount of carbohydrate. Participants ingested their assigned treatment twice daily for 2 days during recovery following an acute bout of eccentric exercise (described below). During the study, participants were required to report to the laboratory on 3 sequential days for testing (not including the visit for preliminary testing and familiarization). Testing occurred at baseline (PRE), immediately post-exercise (POST), 24-h post-exercise (24 h), and 48-h post-exercise (48 h) and consisted of the following: tests of muscle function [maximal isometric voluntary contraction (MIVC) of the knee-extensors and counter movement jump height (CMJ)], muscle soreness [VAS questionnaire and pressure pain threshold (PPT)], markers of exercise induced muscle damage [serum creatine kinase (CK)], limb girth of the upper leg and calf (as a proxy for swelling/edema), flexibility of the quadriceps (via goniometer during a Modified Thomas Test), and plasma concentrations of select cytokines and chemokines. Testing also consisted of the Brief Assessment of Mood Adapted (BAM+) questionnaire to assess the participants “readiness” for performance (23). The randomization procedure to allocate treatment group was determined via a random-number generator (http://www.randomization.com/). An equal number of men and women were randomized to the KET and CON groups, respectively. An independent person was responsible for the randomization and preparation of the study beverages. The beverages were prepared in non-transparent plastic containers. To limit diurnal and intrasubject variation, all measures were carried out according to a standardized time schedule at the same time of day.

### Experimental Protocol

**Figure 1:**
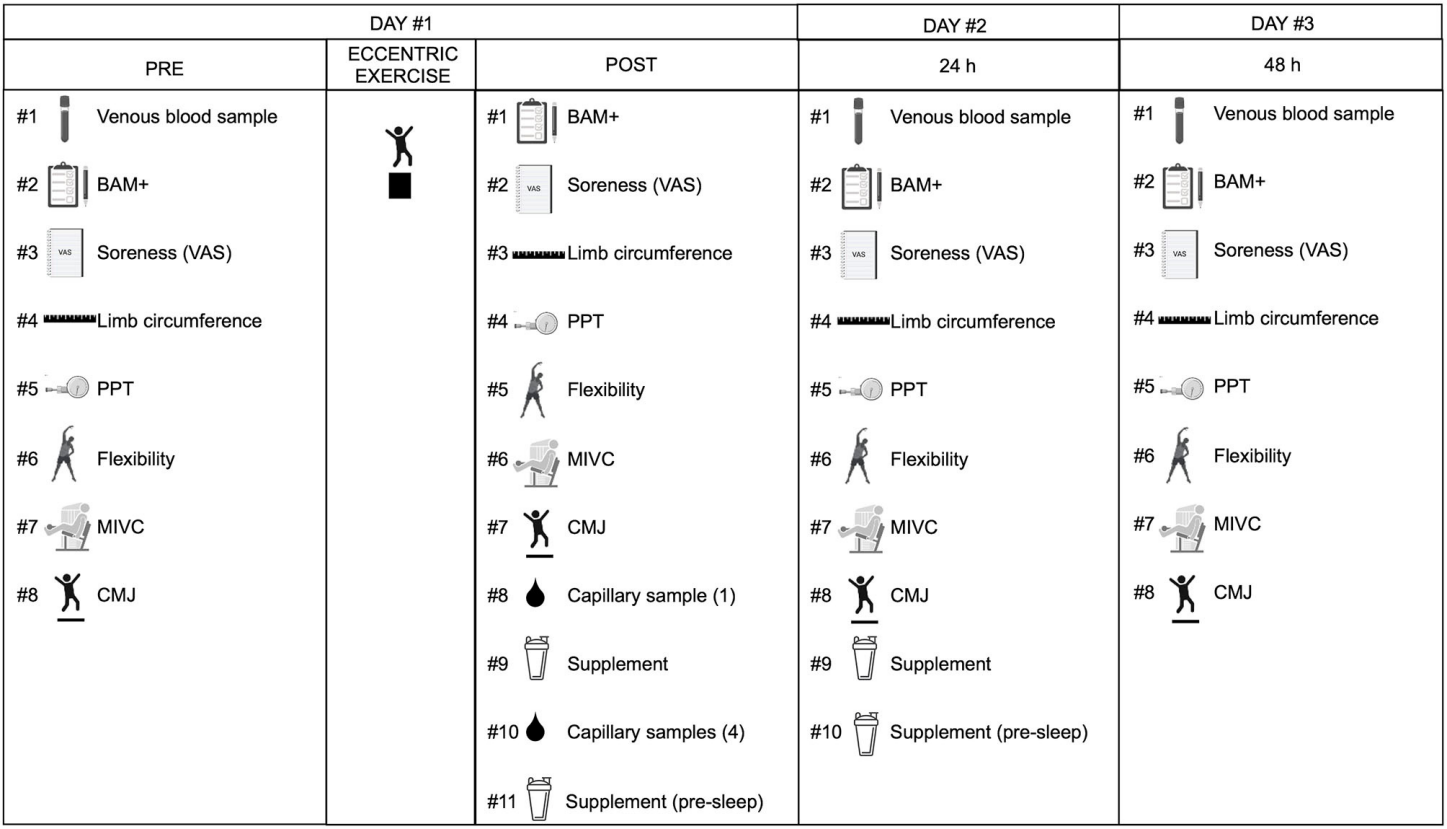
Overview of the experimental protocol. Twenty young adults (10 men; 10 women) performed an acute bout of eccentric exercise and were studied over 48 h of post-exercise recovery. During the recovery period, participants received a ketone monoester (KET) or energy-matched carbohydrate (CON) supplement two times per day (in the morning immediately after testing and in the evening 30 min prior to sleep). Testing occurred at baseline (PRE) before the bout of eccentric exercise, and then immediately (POST), 24 h (24 h), and 48 h (48 h) after exercise and included the following: venous blood sample (except at POST), BAM+, VAS muscle soreness, limb (thigh and calf) circumference, PPT, flexibility, MIVC, and CMJ. Capillary blood samples were taken before (0 min) and after (30, 60, 120, 180 min) intake of the first supplement serving. BAM+, brief assessment of mood adapted; CMJ, counter-movement jump; CON, carbohydrate control group; KET, ketone monoester group; MIVC, maximal isometric voluntary contraction; PPT, pressure-pain threshold; VAS, visual analog scale. Figure created with BioRender.com.

On Day #1 of the study, participants reported to the laboratory at ~08:00 in the overnight post-absorptive state. Participants underwent a venous blood draw from the antecubital vein via venipuncture followed by baseline (PRE) testing in the following order: BAM+ questionnaire, VAS of muscle soreness, limb circumference, PPT, and Modified Thomas Test (measures described below). Subsequently, participants performed a 5 min brisk walking warm-up on a treadmill at a speed of 5.0 km/h with an incline of 1.0, followed by assessment of knee extensor MIVC torque (Biodex 4 Pro, Biodex Medical Systems, Shirley, NY, USA) and CMJ height (Dual force plates model 9260AA6, Kistler group, Winterthur, Switzerland). Participants then performed an eccentric exercise protocol consisting of 100 drop jumps off a 60 cm box (5 sets of 20 repetitions with a 2 min inter-set rest interval). Following 15 min of recovery from the eccentric exercise protocol, participants completed post-exercise testing (POST) of the following: BAM+ questionnaire, VAS of muscle soreness, limb circumference, PPT, Modified Thomas Test, knee extensor MIVC, and CMJ. Participants then received and ingested their randomly assigned treatment (KET or CON). Capillary blood samples were obtained via finger prick to assess capillary blood β-OHB concentration (FreeStyle Precision Neo, Abbott Laboratories) immediately following POST testing prior to treatment intake, and at 30, 60, 120, and 180 min following treatment intake while participants rested in a recumbent position on a bed in the laboratory. An independent person obtained the capillary samples to allow the study investigators to remain blinded to the treatment intervention. Participants then received a second treatment drink that they were instructed to consume on their own in the evening ~30 min before going to sleep. The following morning (Day #2) participants reported to the laboratory in the overnight post-absorptive state and completed the 24 h post-exercise (24 h) testing in a manner identical to PRE (baseline) testing. Participants then received and ingested the third serving of their randomly assigned treatment and returned the containers from the treatment consumed the evening prior. Participants then received their fourth and final treatment drink that they were instructed to consume on their own in the evening ~30 min before going to sleep. The following morning, participants again reported to the laboratory (Day #3) in the overnight post-absorptive state, completed the 48 h post-exercise (48 h) testing protocol (identical to the 24 h testing outlined above), and returned the containers from the treatment consumed the evening prior. For an overview of the experimental protocol see Figure 1.

### Venous Blood Sampling and Analysis

Venous blood samples were drawn into a 4-mL vacutainer for serum and two 3-mL vacutainers coated with Ethylenediaminetetraacetic acid (EDTA) for plasma during PRE, 24 h and 48 h time-points. Samples were centrifuged at 3,000 g for 15 min at 4°C. Plasma and serum were then aliquoted and stored at −80°C until further analysis. Analysis of total CK concentration was carried out on serum samples using a chemistry analyzer (Beckman Coulter Olympus AU5800) at the Clinical Biochemistry Laboratory of the McGill University Health Centre. According to data provided by the laboratory, the coefficient of variation (CV) for total CK is 2.5%. Analysis of circulating granulocyte colony stimulating factor (G-CSF), interleukin-1 beta (IL-1β), interleukin-1 receptor antagonist (IL-1ra), interleukin-6 (IL-6), interleukin-8 (IL-8), interleukin-10 (IL-10), interleukin-12p70 [IL-12(p70)], interleukin-15 (IL-15), monocyte chemoattractant protein 1 (MCP-1), and tumor necrosis factor alpha (TNF-α) was carried out on plasma samples using Luminex xMAP technology for multiplexed quantification of human cytokines, chemokines, and growth factors. The multiplexing analysis was performed in duplicate using the Luminex™ 200 system (Luminex, Austin, TX, USA) by Eve Technologies Corp (Calgary, Alberta). In the first multi-plex experiment, six markers were simultaneously measured in the samples using Eve Technologies' Custom Human High Sensitivity 6-Plex Discovery Assay® (MilliporeSigma, Burlington, Massachusetts, USA) according to the manufacturer's protocol. The 6-plex consisted of IL-6, IL-8, IL-10, IL-12p70, Il-1β, and TNF-α. Assay sensitivities of these markers are 0.11, 0.13, 0.56, 0.15, 0.14, and 0.16 pg/mL, respectively. In the second multiplex experiment, 4 markers were simultaneously measured in the samples using Eve Technologies' Custom Human Cytokine Panel 1 4-Plex (MilliporeSigma, Burlington, Massachusetts, USA) according to the manufacturer's protocol. The 4-plex consisted of IL-1ra, IL-15, MCP-1, and G-CSF. Assay sensitivities of these markers are 8.3, 1.2, 1.9, and 1.8 pg/mL, respectively. At least one of the two duplicate samples were below the limit of analytical sensitivity in 35% of samples for G-CSF, 42% of samples for IL-1ra, and 47% of samples for IL-15. Therefore, these analytes were removed from analysis. The CV's for the analyzed data were as follows: IL-1β: 9.3%; IL-6: 7.0%; IL-8: 9.5%; IL-10: 8.8%; IL-12p70: 8.4%; MCP-1: 5.7%, and TNF-α 7.8%.

### Subjective Mood Questionnaire (BAM+)

The Brief Assessment of Mood Adapted (BAM+) questionnaire was used to qualitatively assess participants' mood, recovery status and overall performance readiness (23). The questionnaire contains six items from The Brief Assessment of Mood (BAM) and four questions relating to confidence, motivation, muscle soreness and sleep quality. The BAM+ consists of ten questions and participants are asked to mark a line on a 100 mm VAS, anchored with “not at all” and “extremely” at each end. The lines for each question were measured with a ruler, and an overall recovery score was calculated as described by Shearer et al. (23).

### Muscle Soreness

Muscle soreness was assessed via VAS questionnaire and determination of PPT. For assessment of muscle soreness using the VAS questionnaire, participants were asked to perform a squat (at ~90° knee flexion) and rate their level of perceived muscle soreness in their lower limbs by marking a 100 mm horizontal line scale. 0 on the VAS represented “no soreness” and 100 mm represented “unbearably painful.” The line placement was measured with a ruler and recorded. The inter-day CV for this measure in our laboratory is 8.3%.

PPT was assessed using a handheld digital algometer (Somedic SenseLAB AB, Sodala, Sweden) at three pre-marked sites: the *vastus lateralis* (VL), *rectus femoris* (RF), and *gastrocnemius*. The VL was assessed mid-way between the superior aspect of the greater trochanter and head of the tibia. The RF was assessed mid-way between the anterior patella and inguinal fold. The gastrocnemius was assessed on the medial aspect of the calf at relaxed maximum girth. The sites were marked with semi-permanent ink to ensure consistent measurements between days. PPT was assessed while participants lay supine on a table by a study investigator applying pressure at the sites with the algometer until the participant indicated they felt the sensation of pressure transition to the feeling of pain, at which point the pressure value in kPa was recorded. Each site was assessed twice unless recordings differed by 100 or more kPa, in which case the site was assessed a third time and the average was recorded. The reported CV for this measure using these three sites is 9.5% (24).

### Limb Circumference

Circumference at the mid-thigh and calf was assessed as a proxy of limb swelling using an anthropometric tape measure. Both sites were measured with the participant in a standing position. The mid-thigh measure was determined as the mid-point between the inguinal crease and superior aspect of the patella. The calf measurement was made at the widest part of the calf. Both sites were marked with semi-permanent ink to ensure consistent measurements between days (25, 26). The inter-day CV for this measure in our laboratory is 1.0%.

### Flexibility

The Modified Thomas test was used to assess flexibility about the thigh region (including hip and knee joint) using a goniometer. The test was performed by having the participant lay supine on the edge of a treatment table, hold his or her non-testing knee to the chest, while letting the opposite (tested) thigh and leg hang freely off the table. The goniometer was lined up to the greater trochanter, the lateral epicondyle of the femur and the lateral malleolus. Hip extension angle (X° – 90°) and knee flexion angle (180° – X°) were then determined. The inter-day CV for this measure in our laboratory is 1.0%.

### Maximal Isometric Voluntary Contraction (MIVC)

MIVC of the knee extensors were assessed using a dynamometer (Biodex 4 Pro, Biodex Medical Systems, Shirley, NY, USA). Participants were seated in an upright position, securely fastened with the knee angle set at 90° and instructed to perform a maximal force knee extension for 3 s. Each participant performed five contractions separated by 1 min of rest. Force was recorded in Torque (Nm). The peak value from five maximal contractions was used for analysis. The inter-day CV for this measure in our laboratory is 5.1%.

### Counter Movement Jump (CMJ)

A force plate system (Dual force plates model 9260AA6, Kistler group, Winterthur, Switzerland) was used to measure CMJ height (cm). Participants were required to descend into a squat position (to a ~90° knee angle) before jumping vertically with maximum effort. During testing, participants' hands remained on the hips. Each participant performed three maximal effort jumps separated by 30 s of rest. The average value of the three jumps was used for analysis. The inter-day CV for this measure in our laboratory is 1.8%.

### Capillary Blood Samples

β-OHB concentration in capillary blood was determined using a handheld monitor (FreeStyle Precision Neo, Abbott Laboratories, Witney, UK). Fingertip capillary samples were collected using a lancet following cleaning with alcohol and allowing to air dry. The first blood droplet sample was discarded with a cotton swab and the subsequent droplet samples were used for analysis. Capillary blood β-OHB concentration was assessed immediately following POST testing prior to treatment intake, and at 30, 60, 120, and 180 min after treatment intake.

### Eccentric Exercise Protocol

The eccentric bout of exercise consisted of 100-drop jumps from a 60 cm high box with a non-slip rubber top (Northern Lights Fitness Products Inc. Cornwall, ON, Canada). Each repetition of the drop jump was performed by having the participant first step onto the box (alternating between legs during each step-up), then “drop” off the box onto the floor and land on two feet, while immediately descending to a ~90° knee angle and jumping vertically with maximal effort. Participants performed five sets of 20 repetitions with a 2-min inter-set rest period. The technique was first demonstrated by one of the study investigators on two occasions before participants performed the exercise bout. Each participant was given corrective feedback and strong verbal encouragement to ensure maximal efforts. Drop jumps have been utilized in several muscle damage research studies and have been demonstrated to induce significant lower limb muscle soreness and declines in muscle function (25, 27–32).

### Nutritional Treatments

The KET group received the ketone monoester (R)-3-hydroxybutyl (R)-3-hydroxybutyrate (Pure ΔG® Ketone Ester; HVMN, CA, USA) at a dose of 360 mg per kg^−1^ body weight per serving. The CON group received an energy-matched amount of carbohydrate as a combination of Glacier Cherry Gatorade (G2—Gatorade Company, Inc., Chicago, IL, USA) and Dextrose powder (NOW foods, Inc., Bloomingdale IL, USA) with 10 drops of liquid Vanilla Stevia (Stevia Select Inc, USA). All treatments were volume-matched and prepared in opaque drinking containers. Participants ingested their assigned treatment twice daily (morning and evening) for 2 days (48 h) during recovery following an acute bout of eccentric exercise (described above). The morning treatments were consumed at the laboratory under direct supervision of one of the study investigators, while participants consumed the evening treatments on their own ~30 min before going to bed. Participants were asked to return the used bottles at the end of the study.

### Statistical Analysis

Due to technical issues, analysis of plasma cytokine/chemokine concentration was not performed in two subjects, therefore plasma cytokine/chemokine data represent *n* = 9 per group. Participants' physical characteristics, baseline (PRE) test results (for MIVC, CMJ, VAS, PPT, limb circumference, flexibility, and BAM+), and peak capillary blood β-OHB concentration were compared with independent sample *t*-tests. The peak response (i.e., peak increase or decrease), calculated as the peak change from baseline (PRE) during recovery after eccentric exercise was compared with independent sample *t*-tests. All other dependent variables were tested using a 2-factor (group × time) repeated-measures ANOVA. When a statistically significant main effect for time or group × time interaction was observed, Bonferroni-corrected *post-hoc* comparisons were performed. Assumptions of the ANOVA models were assessed using Mauchley's test and the D'Agostino–Pearson omnibus normality test at a significance of *P* < 0.05. If a significant Mauchley's test was determined, the Greenhouse-Geisser correction factor was used to adjust the degrees of freedom accordingly. For data that did not pass normality, values were transformed with the ln of the value. The statistical analysis was performed on transformed data, but non-transformed data are presented in graphic or tabular form for clarity. Sample size was determined by completing a power analysis (power = 0.9, α = 0.05) based on differences in isometric strength (MIVC) data from Bowtell et al. (33) during recovery from eccentric muscle damaging exercise. This determined a sample size of 6 in each group would provide statistical power at 90%, with an alpha level of 0.05. However, to preserve power and account for dropouts, we recruited *n* = 10 per group. Statistical analysis was performed with use of the Statistical Package for the Social Sciences (SPSS, Version 24. IBM Corp., Armonk, NY, USA) and GraphPad Prism (Version 8. GraphPad Software., San Diego, CA, USA). For all analyses, differences were considered statistically significant at *P* < 0.05. All data are expressed as means ± SD.

## Results

### Participants' Characteristics and Baseline Test Scores

There were no differences between KET and CON groups for the participants' characteristics or baseline (PRE) test scores (Table 1).

### Dietary Intake

Average total energy, fat, and protein intake, as well as protein intake relative to bodyweight were not different between the pre-study phase compared to the EIMD recovery phase (all *P* > 0.05) and were not different between KET and CON groups (*P* > 0.05). Average carbohydrate intake was lower during the EIMD recovery phase compared to the pre-study phase (time; *P* = 0.03) but was not different between KET and CON groups (*P* > 0.05). Average dietary intake of study participants is shown in Table 2.

**Table 2:** Average dietary intake of study participants over 2 days prior to the onset of the experiment (pre-study phase) and during the experiment (EIMD recovery phase) who ingested a ketone monoester supplement (KET) or energy-matched carbohydrate supplement (CON).

	**KET (*****n*** **=** **10)**		**CON (*****n*** **=** **10)**	
	**Pre-study phase**	**EIMD recovery phase**	**Pre-study phase**	**EIMD recovery phase**
Energy, kcal·d^−1^	2548, 941	2319, 634	2464, 831	2020, 565
Carbohydrate, g	334, 162^a^	276, 98^b^	289, 99^a^	193, 56^b^
Fat, g	88, 37	91, 37	98, 35	96, 39
Protein, g	107, 35	104, 27	112, 49	114, 43
Protein, g·kg^−1^·d^−1^	1.5, 0.6	1.5, 0.5	1.6, 0.6	1.6, 0.5

*Values represent mean ± SD. Phases without a common letter differ. Data were analyzed with a two-factor repeated measures (within subject factor: time; between-subject factor: group) ANOVA. Energy, kcal· d^−1^; time effect: P = 0.053; group effect: P = 0.526; group × time interaction: P = 0.518. Carbohydrate, g; time effect: P = 0.03; group effect: P = 0.124; group × time interaction: P = 0.263. Fat, g; time effect: P = 0.934; group effect: P = 0.644; group × time interaction: P = 0.762. Protein, g; time effect: P = 0.875; group effect: P = 0.646; group × time interaction: P = 0.670. Protein, g·kg^−1^·d^−1^; time effect: P = 0.848; group effect: P = 0.753; group × time interaction: P = 0.619. CON, carbohydrate control group; KET, ketone monoester group*.

### Capillary Blood β-OHB Concentration

**Figure 2:**
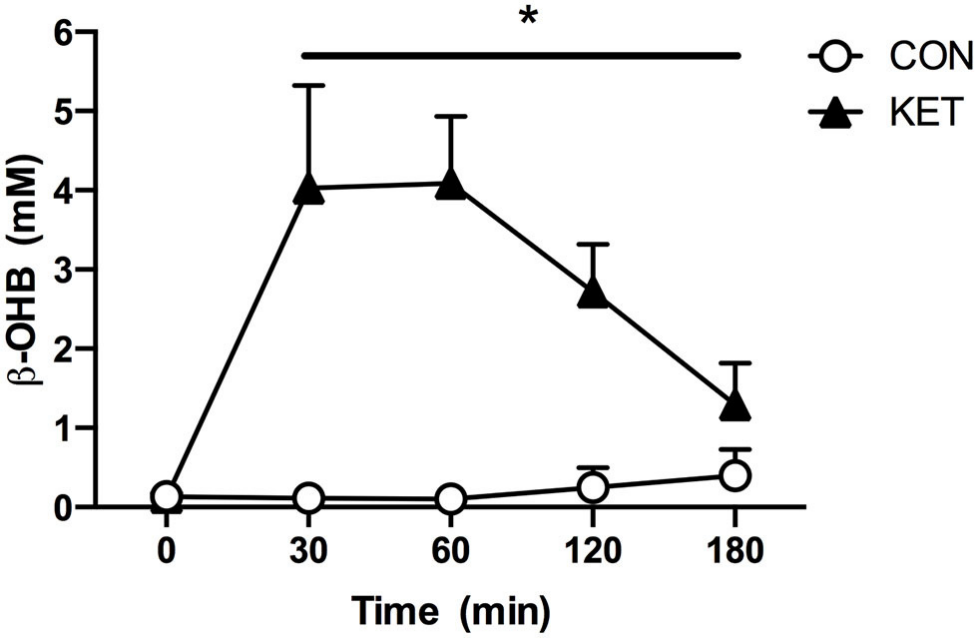
Capillary blood β-OHB concentration (mM) immediately following POST testing prior to treatment intake (*t* = 0 min) and 30, 60, 120, and 180 min after treatment intake. Values represent means ± SD. *Indicates difference between KET and CON. Data were analyzed with a two-factor repeated measures (within subject factor: time; between-subject factor: group) ANOVA. Bonferroni-corrected *post-hoc* comparisons were performed following a significant main effect for time or group × time interaction. Time effect: *P* < 0.001; group effect: *P* < 0.001; group × time interaction: *P* < 0.001. β-OHB, beta-hydroxybutyrate; CON, carbohydrate control group; KET, ketone monoester group.

Capillary blood β-OHB concentration (Figure 2) increased (group × time interaction; *P* < 0.001) following ingestion of the ketone monoester and was significantly higher in KET compared to CON at 30 (KET: 4.0 ± 1.3; CON: 0.1 ± 0.03 mM), 60 (KET: 4.1 ± 0.8; CON: 0.1 ± 0.0 mM), 120 (KET: 2.7 ± 0.6; CON: 0.3 ± 0.2 mM), and 180 (KET: 1.3 ± 0.5; CON: 0.4 ± 0.3 mM) min after intake (all *P* < 0.001).

### Serum Creatine Kinase Concentration

**Figure 3:**
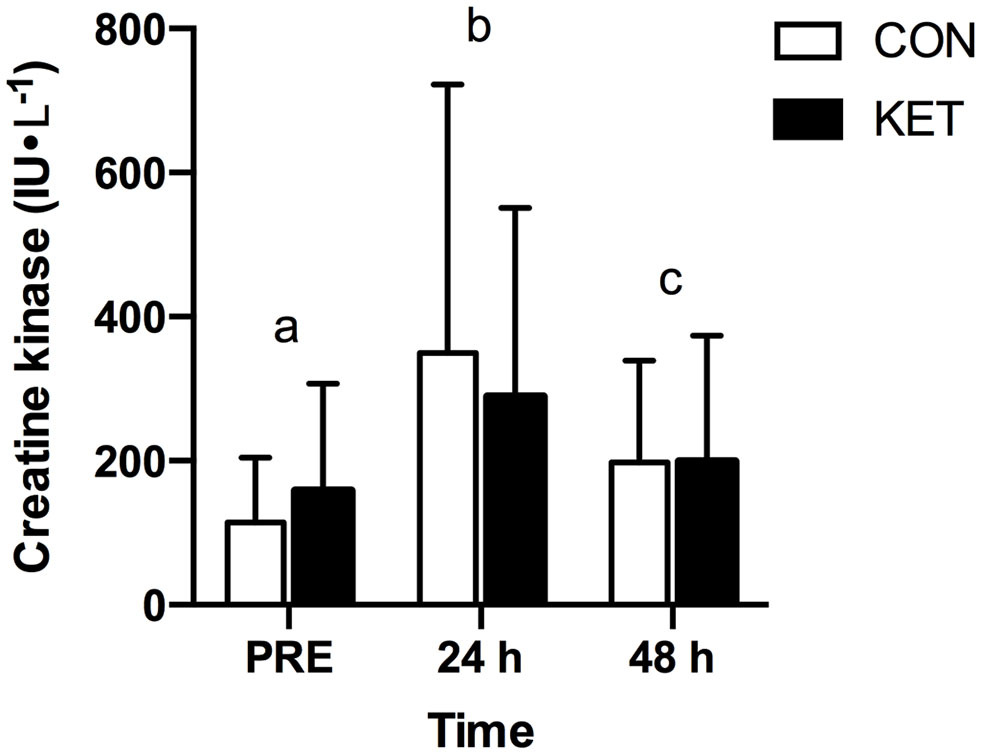
Serum creatine kinase concentration (IU·L^−1^) at baseline (PRE) prior to exercise, and 24 h and 48 h after exercise. Values represent means ± SD. Times without a common letter differ. Data were analyzed with a two-factor repeated measures (within subject factor: time; between-subject factor: group) ANOVA. Bonferroni-corrected *post-hoc* comparisons were performed following a significant main effect for time or group × time interaction. Time effect: *P* < 0.001; group effect: *P* = 0.918; group × time interaction: *P* = 0.313. CON, carbohydrate control group; KET, ketone monoester group.

Serum CK concentration (Figure 3) was increased (time; *P* < 0.001) at 24 h (319 ± 315 IU·L^−1^; *P* < 0.001) and 48 h (198 ± 155 IU·L^−1^; *P* = 0.037) after eccentric exercise when compared to PRE (136 ± 122 IU·L^−1^). There were no differences between KET and CON (group; *P* = 0.918). The peak increase in CK concentration from PRE after eccentric exercise was not different between groups (KET: 162 ± 195; CON: 235 ± 385 IU·L^−1^; *P* = 0.595).

### Maximal Isometric Voluntary Contraction and Counter Movement Jump Height

**Figure 4:**
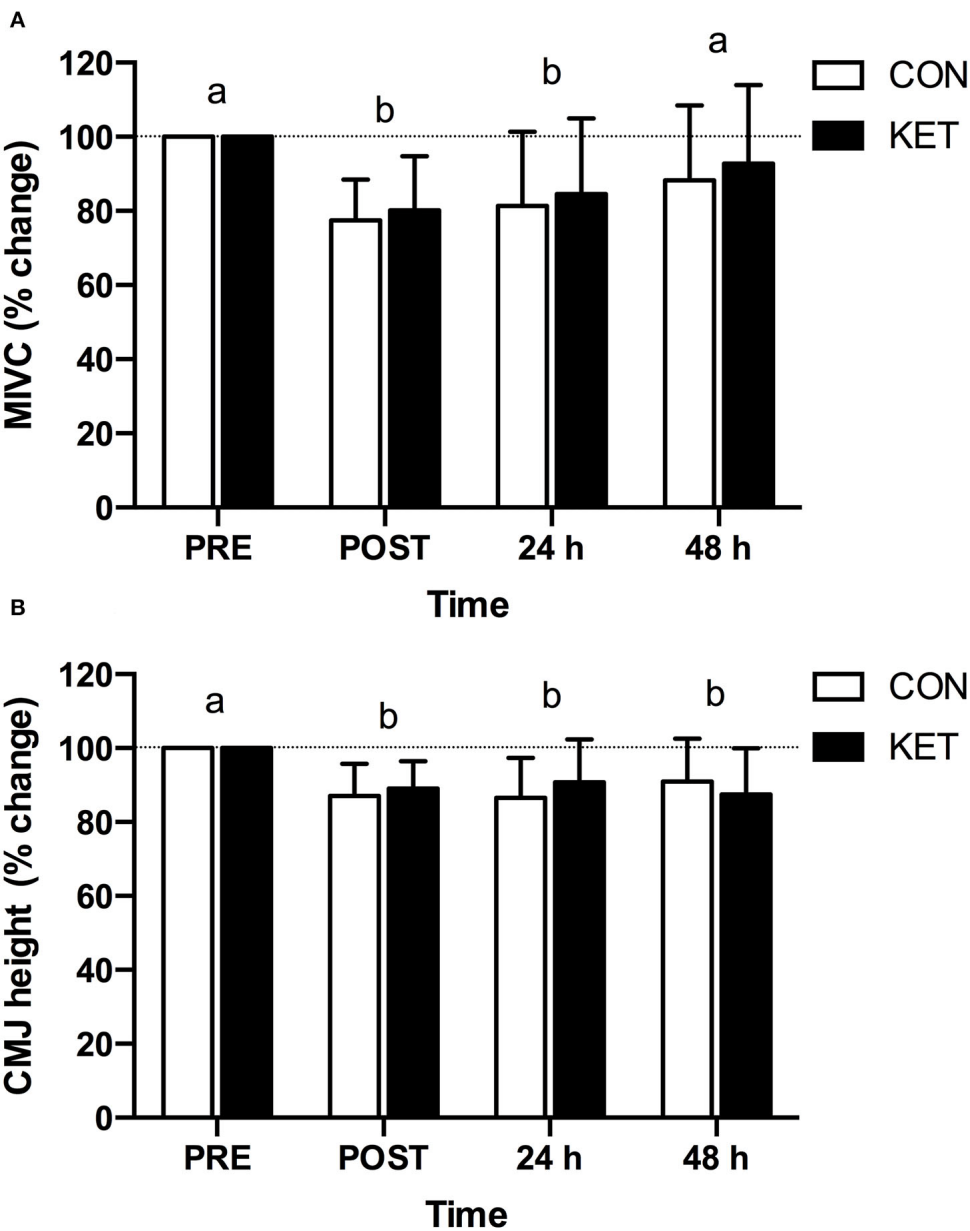
Percent change in maximal isometric voluntary contraction **(A)** and counter-movement jump **(B)** relative to baseline (PRE) prior to exercise, and immediately (POST), 24 h and 48 h after exercise. Values represent means ± SD. Times without a common letter differ. Data were analyzed with a two-factor repeated measures (within subject factor: time; between-subject factor: group) ANOVA. Bonferroni-corrected *post-hoc* comparisons were performed following a significant main effect for time or group × time interaction. Maximal isometric voluntary contraction; time effect: *P* < 0.001; group effect: *P* = 0.612; group × time interaction: *P* = 0.594. Counter-movement jump; time effect: *P* < 0.001; group effect: *P* = 0.571; group × time interaction: *P* = 0.378. CMJ, counter-movement jump; CON, carbohydrate control group; KET, ketone monoester group; MIVC, maximal isometric voluntary contraction.

MIVC torque (Figure 4A) was decreased (time; *P* < 0.001) at POST (−21.2 ± 12.7%; *P* < 0.001) and 24 h (−17.1 ± 19.8%; *P* = 0.003) compared to PRE following eccentric exercise. There were no differences in MIVC between KET and CON (group; *P* = 0.612). The peak decline in absolute MIVC torque from PRE during recovery after eccentric exercise was not different between groups (KET: −43.7 ± 26.0; CON: −54.6 ± 28.3 Nm; *P* = 0.497). Counter movement jump height (Figure 4B) was decreased (time; *P* < 0.001) at POST (−12.0 ± 8.0%; *P* < 0.001), 24 h (−11.3 ± 11.1%; *P* = 0.003), and 48 h (−10.8 ± 11.9%; *P* = 0.010) compared to PRE after eccentric exercise. There were no differences in CMJ between KET and CON (group; *P* = 0.571). The peak decline in absolute CMJ height from PRE during recovery after eccentric exercise was not different between groups (KET: −5.2 ± 3.8; CON: −4.9 ± 2.5 cm; *P* = 0.875).

### Muscle Soreness and Pressure Pain Threshold

**Figure 5:**
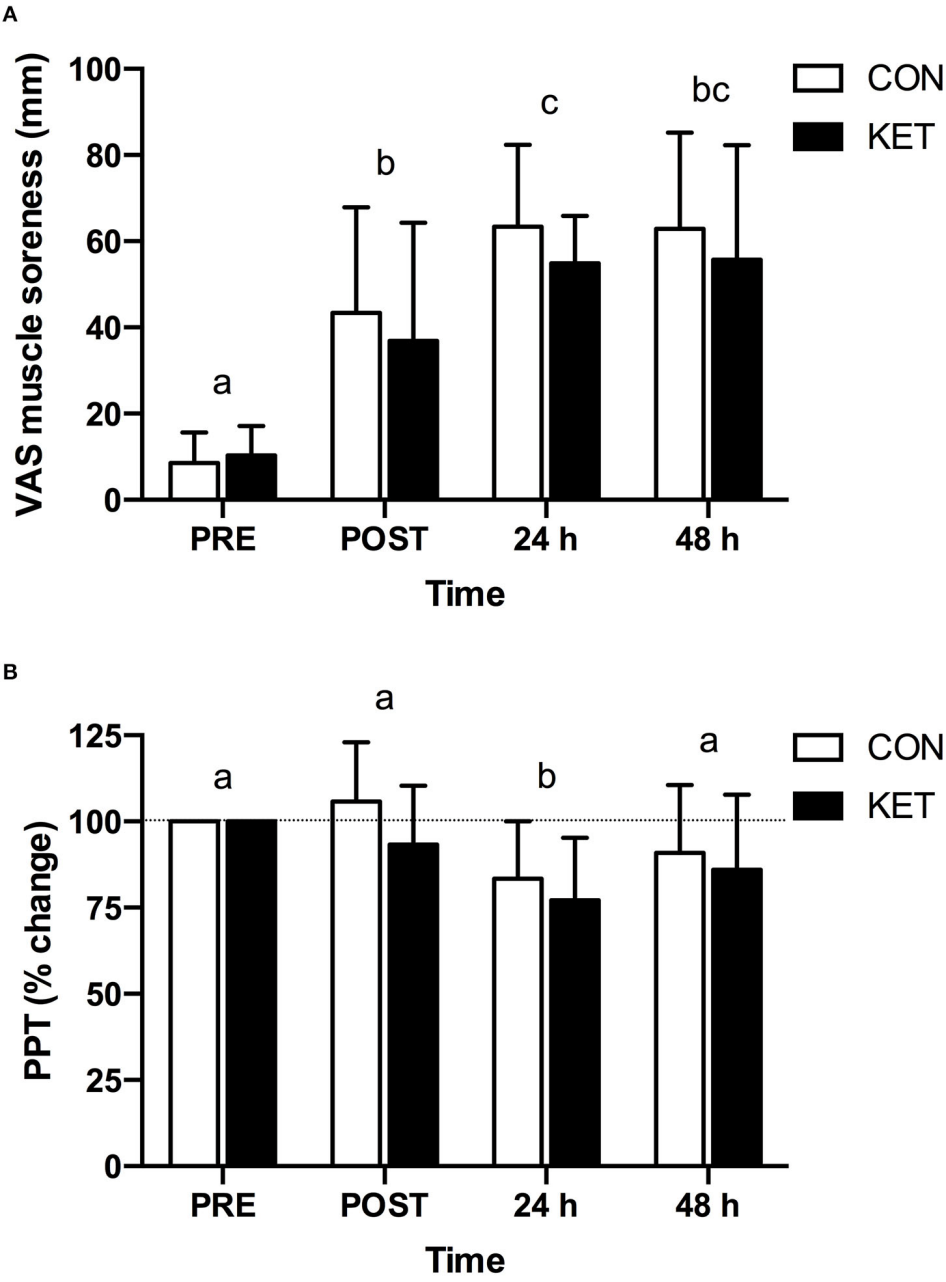
Visual analog scale derived muscle soreness **(A)** and percent change in pressure-pain threshold **(B)** at or relative to baseline (PRE) prior to exercise, and immediately (POST), 24 h and 48 h after exercise. Values represent means ± SD. Times without a common letter differ. Data were analyzed with a two-factor repeated measures (within subject factor: time; between-subject factor: group) ANOVA. Bonferroni-corrected *post-hoc* comparisons were performed following a significant main effect for time or group × time interaction. Visual analog scale derived muscle soreness; time effect: *P* < 0.001; group effect: *P* = 0.896; group × time interaction: *P* = 0.517. Pressure-pain threshold; time effect: *P* < 0.001; group effect: *P* = 0.113; group × time interaction: *P* = 0.492. CON, carbohydrate control group; KET, ketone monoester group; PPT, pressure-pain threshold; VAS, visual analog scale.

Ratings of muscle soreness (Figure 5A) were increased (time; *P* < 0.001) after eccentric exercise at POST, 24 h, and 48 h compared to PRE (all *P* < 0.001). Ratings of muscle soreness were also greater at 24 h (*P* = 0.010) compared to POST. There were no differences in ratings of muscle soreness between KET and CON (group; *P* = 0.896). The peak increase in absolute ratings of muscle soreness from PRE during recovery after eccentric exercise was not different between groups (KET: 53 ± 18; CON: 59 ± 20 cm; *P* = 0.946). The PPT (Figure 5B) was reduced (time; *P* < 0.001) at 24 h (*P* = 0.002) following eccentric exercise compared to PRE. There were no differences in PPT between KET and CON (group; *P* = 0.113). The peak decline in absolute PPT from PRE during recovery after eccentric exercise was not different between groups (KET: −129 ± 154; CON: −62 ± 39 kPa; *P* = 0.276).

### Thigh Circumference, Calf Circumference, and Flexibility

Thigh circumference (see Supplementary Figure 1A) was increased (time; *P* < 0.001) at POST (*P* < 0.001) and 24 h (*P* = 0.015) compared to PRE. There were no differences in thigh circumference between KET and CON (group; *P* = 0.584). The peak increase in absolute thigh circumference from PRE during recovery after eccentric exercise was not different between groups (KET: 1.4 ± 0.9; CON: 1.4 ± 0.8 cm; *P* = 1.000). Calf circumference (see Supplementary Figure 1B) was increased (time; *P* = 0.001) at POST compared to PRE (*P* < 0.001) following the bout of eccentric exercise. There were no differences in calf circumference between KET and CON (group; *P* = 0.771). The peak increase in absolute calf circumference from PRE during recovery after eccentric exercise was not different between groups (KET: 0.6 ± 0.4; CON: 0.6 ± 0.4 cm; *P* = 0.791). Flexibility (see Supplementary Figure 1C) was not different over time (time; *P* = 0.428) or between KET and CON (group; *P* = 0.594).

### Brief Assessment of Mood Adapted

BAM+ scores (see Supplementary Figure 2) were reduced (time; *P* = 0.001) at POST (*P* = 0.011) and 24 h (*P* = 0.008) following the bout of eccentric exercise compared to PRE. There were no differences in BAM+ scores between KET and CON (group; *P* = 0.793). The peak decline in BAM+ from PRE during recovery after eccentric exercise was not different between groups (KET: −28.1 ± 25.0; CON: −33.4 ± 22.7 AU; *P* = 0.626).

### Plasma Cytokines and Chemokines

Plasma cytokines and chemokines are shown in Table 3. Plasma IL-1β showed a group × time interaction (*P* = 0.027); however, Bonferroni corrected pairwise comparisons showed no differences between KET and CON at any time-point (all *P* > 0.05). Plasma IL-6 was not different over time (time; *P* = 0.650) or between KET and CON (group; *P* = 0.166). Plasma IL-8 was not different over time (time; *P* = 0.677) or between KET and CON (group; *P* = 0.998). Plasma IL-10 was not different over time (time; *P* = 0.259) or between KET and CON (group; *P* = 0.594). Plasma IL-12(p70) was not different over time (time; *P* = 0.185) or between KET and CON (group; *P* = 0.287). Plasma MCP-1 was greater in CON compared to KET (group; *P* = 0.007). Plasma TNF-α was not different over time (time; *P* = 0.619) or between KET and CON (group; *P* = 0.153).

**Table 3:** Plasma IL-1β, IL-6, IL-8, IL-10, IL-12(p70), MCP-1, and TNF-α concentration at baseline (PRE) prior to exercise, and 24 h and 48 h after exercise in participants who ingested a ketone monoester supplement (KET) or energy-matched carbohydrate supplement (CON).

	**KET (*****n*** **=** **9)**			**CON (*****n*** **=** **9)**		
	**PRE**	**24 h**	**48 h**	**PRE**	**24 h**	**48 h**
IL-1β (pg/mL)	1.8, 1.1	1.9, 0.9	1.8, 0.8	2.3, 1.8	1.6, 0.8	1.8, 0.9
IL-6 (pg/mL)	1.8, 0.7	1.8, 0.6	1.8, 0.4	2.3, 0.8	2.1, 0.5	2.0, 0.4
IL-8 (pg/mL)	5.9, 2.2	6.3, 2.3	6.3, 2.2	6.9, 2.6	5.7, 1.9	5.9, 2.1
IL-10 (pg/mL)	5.1, 2.4	5.1, 2.5	5.2, 2.5	7.5, 7.1	4.9, 2.6	5.3, 2.7
IL-12(p70) (pg/mL)	4.3, 1.0	4.3, 1.1	4.3, 1.4	4.9, 4.2	3.4, 1.3	3.5, 1.3
MCP-1 (pg/mL)^*^	134.1, 28.6	125.2, 21.0	131.9, 52.1	155.0, 21.9	196.7, 63.7	153.0, 25.7
TNF-α (pg/mL)	6.1, 1.3	5.8, 1.2	5.7, 1.3	7.5, 4.3	7.5, 2.3	7.0, 3.0

*Values represent mean ± SD*.

* *Indicates difference between KET and CON. Data were analyzed with a two-factor repeated measures (within subject factor: time; between-subject factor: group) ANOVA. Plasma IL-1β; time effect: P = 0.130; group effect: P = 0.931; group × time interaction: P = 0.027. Plasma IL-6; time effect: P = 0.650; group effect: P = 0.166; group × time interaction: P = 0.317. Plasma IL-8; time effect: P = 0.677; group effect: P = 0.998; group × time interaction: P = 0.145. Plasma IL-10; time effect: P = 0.259; group effect: P = 0.594; group × time interaction: P = 0.232. Plasma IL-12(p70); time effect: P = 0.185; group effect: P = 0.287; group × time interaction: P = 0.241. Plasma MCP-1; time effect: P = 0.253; group effect: P = 0.007; group × time interaction: P = 0.104. Plasma TNF-α; time effect: P = 0.619; group effect: P = 0.153; group × time interaction: P = 0.849. CON, carbohydrate control group; IL-1β, interleukin-1 beta; IL-6, interleukin-6; IL-8, interleukin-8; IL-10, interleukin-10; IL-12(p70), interleukin-12 p70; KET, ketone monoester group; MCP-1, monocyte chemoattractant protein 1; TNF-α, tumor necrosis factor alpha*.

## Discussion

In the current study, we evaluated the impact of twice daily (morning and evening 30 min prior to sleep) supplementation (360 mg per kg^−1^ bodyweight per serving) with a ketone monoester (KET) as compared to an energy-matched carbohydrate control (CON) on indices of EIMD during recovery from a single bout of eccentric exercise in healthy, moderately active, young adults. MIVC was reduced by 19.9 ± 14.6 and 22.6 ± 11.1%, while CMJ was reduced by 11.0 ± 7.5 and 13.0 ± 8.7% at POST in KET and CON groups, respectively, following eccentric exercise. Acute ingestion of the ketone monoester supplement following POST testing markedly elevated circulating blood β-OHB concentration from 30 to 180 min after intake, demonstrating acute nutritional ketosis in the KET group. However, twice daily ketone monoester supplementation did not alter the exercise-induced increase in serum CK concentration, or expedite the recovery of MIVC or CMJ at 24 h or 48 h after exercise compared to CON. Similarly, there were no differences between KET and CON on measures of muscle soreness (VAS and PPT), limb girth, flexibility, BAM+, or the concentration of select pro- and anti-inflammatory cytokines. Overall, twice daily ingestion of a ketone monoester supplement that acutely elevates blood β-OHB concentration does not expedite the recovery of muscle performance or reduce muscle soreness following eccentric exercise in moderately active, healthy young adults.

Consistent with previous work (10), acute ingestion of the ketone monoester (KET) led to a rapid (within 30 min) and pronounced (peak: 4.4 ± 0.8 mM) increase in blood β-OHB concentration that was sustained for at least 180 min (Figure 2). Changes in blood β-OHB concentration were assessed in response to initial (first) supplement intake on Day 1 following POST testing; blood β-OHB concentration following supplement intake in the evening was not assessed as participants were instructed to ingest their respective treatment at home ~30 min prior to sleep. It has previously been reported that ketone monoester intake increases the blood β-OHB: acetoacetate concentration in a 5:1 ratio (34). Therefore, peak total blood ketone body concentrations of ~5.3 mM may have been achieved post-exercise; concentrations similar to those achieved with fasting (35).

Consistent with other studies utilizing the same exercise protocol (25, 31, 32), drop-jump exercise resulted in increases in serum CK concentration when assessed at 24 h and 48 h post-exercise (Figure 3). It has been suggested that a ketogenic diet may reduce EIMD based on the observation that blood CK was reduced 24 h post-exercise in rodents fed a ketogenic vs. control diet (36). Although both exogenous ketone supplements and a ketogenic diet both increase circulating blood ketone body concentration, they exert two distinct metabolic states (37–39) and may therefore have different effects on post-exercise recovery and/or performance. For example, ketone monoester ingestion can increase circulating β-OHB 3–5 mM within ~30 min without requiring carbohydrate restriction (11), whereas achieving nutritional ketosis via a ketogenic diet may take days to weeks and requires dietary carbohydrate restriction (37, 38). The lack of difference between KET and CON on serum CK concentration in the present study suggests that ketone monoester supplementation after exercise might not be beneficial for attenuating damage to the cell membrane resulting from eccentric exercise.

The decline in muscle force production following eccentric exercise is considered one of the most reliable indirect markers of EIMD (40, 41). Although not unequivocal, a number of nutritional interventions including use of branched-chain amino acids, protein, creatine, omega-3 polyunsaturated fatty acids, vitamin D, beetroot, pomegranate, and cherries have been reported to expedite impaired force production and/or performance after muscle damaging exercise [for reviews see (2, 5–7)]. In the present study, we hypothesized that ketone monoester supplementation and elevated β-OHB may accelerate the recovery of indices of muscle damage via mechanisms related to the reported capacity of β-OHB to stimulate protein synthesis (11, 17) and suppress proteolysis (19), and/or to modulate inflammation (42) and oxidative stress (43–45). Eccentric exercise led to a similar decline in MIVC at POST, indicating a similar initial response to the exercise stimulus in both the KET and CON group (Figure 4A). However, the recovery of MIVC did not differ between KET and CON groups when assessed 24 h or 48 h after exercise. Similarly, the eccentric exercise protocol led to an early decline in CMJ height at POST (Figure 4B); however, the recovery of CMJ height was not expedited in KET compared to CON at 24 h or 48 h post-exercise. To our knowledge, this is the first study to evaluate the impact of ketone monoester supplementation as a nutritional strategy to accelerate the recovery of impaired muscle function after eccentric exercise. In terms of the effects of ketone supplements on post-exercise recovery, Vandoorne et al. (11) recently reported that co-ingestion of a ketone monoester with protein and carbohydrate after strenuous exercise enhanced the post-exercise activation of downstream targets of mTORC1 compared to protein and carbohydrate only. Furthermore, ketone bodies increased leucine-mediated protein synthesis rates in C_2_C_12_ cells. However, post-exercise recovery was evaluated for 5 h and changes in muscle performance were not assessed. Huang et al. (36) reported that a ketogenic diet accelerated post-exercise recovery of performance in rodents based on measures of locomotion time taken 24 h after a bout of exhaustive exercise. However, as indicated above, there are substantial differences between a ketogenic diet and ketone supplements (37–39) and the translation of these findings to humans is unclear. Overall, twice daily supplementation with a ketone monoester that acutely raises circulating β-OHB does not expedite the recovery of muscle function based on static (MIVC) or dynamic (CMJ) measures of performance over the initial 2 days after eccentric exercise.

Muscle soreness is a common symptom following eccentric exercise and is the most utilized marker of muscle injury in muscle damage research (40). Recent evidence indicates that intramuscular generation of molecules, such as nerve growth factor, bradykinin, and prostaglandin E2, likely play an important role in muscle soreness due to EIMD (3, 46). These substances can be synthesized by immune cells and act to excite and sensitize local muscle nociceptors, resulting in sensations of pain and soreness in the muscle belly and connective tissues (3). A number of nutritional interventions have been reported to reduce muscle soreness following eccentric exercise [for reviews see (2, 5–7)]. In the present study, muscle soreness was increased in response to eccentric exercise based on both VAS and PPT measures, but there was no difference between KET and CON groups (Figures 5A,B). Therefore, ketone monoester supplementation does not appear to be an effective nutritional strategy to reduce muscle soreness that manifests over the initial 2 days during recovery after eccentric exercise.

Nutritional solutions to expedite the recovery of indices of muscle damage are thought to interact with the secondary damage cascade characterized by inflammation and oxidative stress, to attenuate the exacerbation of damage and aid subsequent recovery (2, 9). Ketone bodies appear to modulate inflammation and immune cell function, with β-OHB exerting a predominantly anti-inflammatory response (20). β-OHB has been reported to lead to anti-inflammatory effects in TNF-α and lipopolysaccharide-induced inflammation by reducing pro-inflammatory proteins, such as inducible nitric oxide synthase and cyclooxygenase-2, or cytokines, such as TNF-α, IL-1β, IL-6, and MCP-1, partly by inhibiting NF-κB (47, 48). In a recent animal study, β-OHB was also shown to decrease inflammation via reducing inflammatory cytokines, such as IL-1β via blocking the NOD-, LRR-, and pyrin domain-containing protein 3 (NLRP3) inflammasome pathway (42). Antioxidant and oxidative stress-mitigating roles of ketone bodies have also been described (20). For example, ketone bodies have been shown to reduce cellular damage, injury, death, and apoptosis in cardiomyocytes and neurons (45, 49, 50), with β-OHB able to scavenge reactive oxygen species, such as hydroxyl anion (49). Recently, β-OHB has been shown to be a histone deacetylase inhibitor, which can increase the expression of antioxidant genes and proteins responsible for decreasing cellular oxidative stress (43). In the present study, we measured plasma concentrations of select pro- and anti-inflammatory cytokines [IL-1β, IL-6, IL-10, IL-12(p70), and TNF-α] and chemokines (IL-8 and MCP-1) at baseline (PRE), 24 h and 48 h post-exercise recovery (Table 3). We observed no difference over time or between KET and CON groups on all markers except MCP-1, which was lower in KET vs. CON (group: *P* = 0.007). This may suggest that (1) the eccentric exercise drop-jump protocol was not damaging enough to induce a systemic inflammatory response, (2) that these systemic markers are not representative of the inflammatory response to eccentric exercise, or (3) that we missed early circulating cytokine responses due to our chosen sampling time-points (i.e., 24 h and 48 h post-exercise). Regarding the first point, previous studies using a similar exercise protocol (51, 52) have reported a marked inflammatory response, including significant elevations in IL-6 up to 24 h post-exercise (52). Differences between these studies and the present investigation may relate to differences in the study population. Regarding the second point, a review by Peake et al. (53) indicated that plasma concentrations of IL-1β, IL-6, IL-8, IL-10, MCP-1, and TNF-α increase in response to exercise. However, plasma cytokine responses with exercise are quite variable between individuals and are dependent on the combination of mode, intensity, and duration of exercise (53). Therefore, we believe there was a strong rationale for examining the plasma concentrations of these cytokines and chemokines with exercise but acknowledge that a different type and/or intensity of exercise may have led to a more robust systemic cytokine response. Regarding the third point, Paulsen et al. (41) mapped the time course for systemic cytokine responses to various forms of eccentric exercise and reported that many systemic cytokine responses occur <24 h post-exercise. Given that the first post-exercise blood sample in the present study was taken 24 h after exercise, we may not have captured early time and or group-dependent differences in circulating cytokines. However, even if we missed any group dependent differences in circulating cytokine concentration that may have occurred early after exercise (<24 h), these were clearly insufficient to augment the recovery of muscle function (MIVC and CMJ) or muscle soreness (VAS and PPT) at 24 h and 48 h post-exercise. To better capture the exercise-induced inflammatory response, early post-exercise blood sample collection, evaluation of additional markers of inflammation, and assessment of intramuscular cytokine mRNA/protein expression and/or inflammatory cell (e.g., neutrophils, macrophages) infiltration (4) may be necessary.

Potential limitations to the present study include the parallel group nature of the study design, choice of eccentric exercise protocol to study indices of EIMD, and utilization of moderately active (regularly exercising 2–4 times per week) research participants. The inter-subject response to EIMD is quite variable (54), and therefore, the absence of a significant difference between the KET and CON group on indices of EIMD may be partly due to the high biological variability of the subjects. While numerous studies have applied box drop exercise within the context of EIMD research (24, 25, 31, 32, 55), the amount of muscle damage experienced by the majority of the research participants in the present study based on the decline in MIVC would be considered mild to moderate (41). Utilization of a more damaging form of eccentric exercise (e.g., unaccustomed isolated maximal eccentric exercise across a large range of motion) and/or utilization of exercise naïve research participants to minimize any potential impact of the repeated bout effect may have resulted in more severe EIMD. Whether ketone monoester supplementation may facilitate recovery following severe EIMD [i.e., eccentric exercise leading to a decline in force-generating capacity >50% and/or recovery of force-generating capacity that exceeds 7 days (41)] is unclear and requires further investigation. More severe EIMD that is accompanied by a greater inflammatory response may represent an environment where any potential benefit of ketone monoester supplementation on post-exercise recovery can be better unmasked.

In summary, results of the present study demonstrate that twice daily ingestion of a ketone monoester supplement that acutely elevates blood β-OHB concentration does not enhance the recovery of muscle performance or reduce muscle soreness following an acute bout of eccentric exercise in moderately active, healthy young adults.

## Data Availability Statement

The raw data supporting the conclusions of this article will be made available by the authors, without undue reservation.

## Ethics Statement

The studies involving human participants were reviewed and approved by Faculty of Medicine Institutional Review Board of McGill University. The patients/participants provided their written informed consent to participate in this study.

## Author Contributions

PM-A and TC-V conceived and designed the research, analyzed the data, interpreted the results of the experiments, prepared the figures, drafted the manuscript, and hold the primary responsibility for the final content. PM-A, JL, and JD conducted the research. PM-A, JM, and TC-V edited and revised the manuscript. All authors read and approved the final manuscript.

## Conflict of Interest

The authors declare that the research was conducted in the absence of any commercial or financial relationships that could be construed as a potential conflict of interest.
