# Supplementary material for: Ketone Monoester Supplementation Does Not Expedite the Recovery of Indices of Muscle Damage After Eccentric Exercise
Source: Front Nutr. 2020 Dec 8;7:607299. doi: 10.3389/fnut.2020.607299 (PMC7752861; doi:10.3389/fnut.2020.607299)
Supplement: Supplementary file 1 [file Data_Sheet_1.PDF]

Supplementary Material

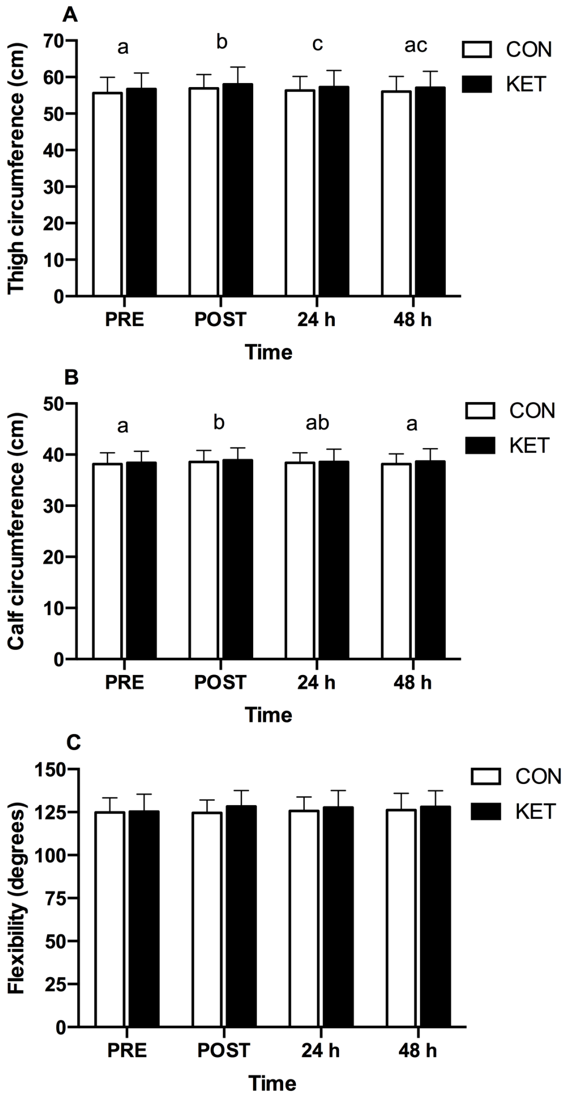

Supplementary Figure 1. Thigh circumference (A), calf circumference (B), and flexibility (C) at baseline (PRE) prior to exercise, and immediately (POST), 24 h, and 48 h after exercise. Values represent means ± SD. Times without a common letter differ. Data were analyzed with a two-factor repeated measures (within subject factor: time; between-subject factor: group) ANOVA. Bonferroni-corrected post hoc comparisons were performed following a significant main effect for time or group × time interaction. Thigh circumference; time effect: $P < 0.001$; group effect: $P = 0.584$; group × time interaction: $P = 0.960$. Calf circumference; time effect: $P = 0.001$; group effect: $P = 0.771$; group × time interaction: $P = 0.443$. Flexibility; time effect: $P = 0.428$; group effect: $P = 0.594$; group × time interaction: $P = 0.603$. CON: carbohydrate control group; KET: ketone monoester group.
