# Supplementary material for: Ketone Monoester Supplementation Does Not Expedite the Recovery of Indices of Muscle Damage After Eccentric Exercise
Source: Front Nutr. 2020 Dec 8;7:607299. doi: 10.3389/fnut.2020.607299 (PMC7752861; doi:10.3389/fnut.2020.607299)
Supplement: Supplementary file 2 [file Data_Sheet_2.PDF]

Supplementary Material

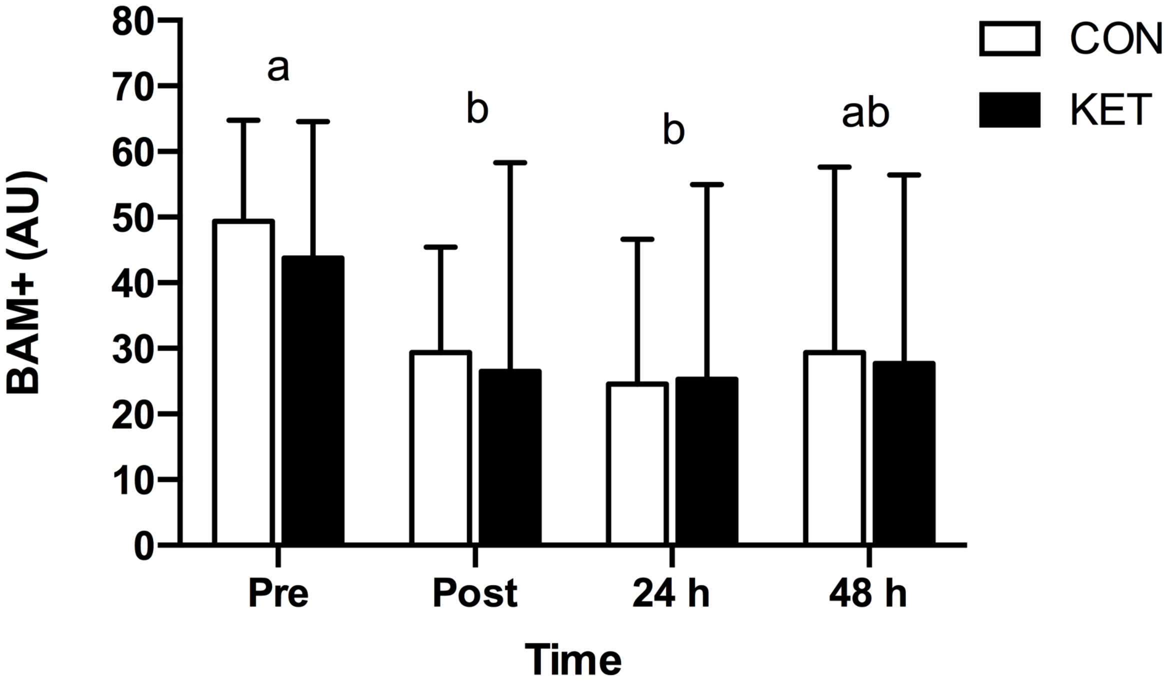

Supplementary Figure 2. Brief assessment of mood adapted at baseline (PRE) prior to exercise, and immediately (POST), 24 h, and 48 h after exercise. Values represent means ± SD. Times without a common letter differ. Data were analyzed with a two-factor repeated measures (within subject factor: time; between-subject factor: group) ANOVA. Bonferroni-corrected post hoc comparisons were performed following a significant main effect for time or group × time interaction. Time effect: P = 0.001; group effect: P = 0.793; group × time interaction: P = 0.945. AU: arbitrary units; BAM+: brief assessment of mood adapted; CON: carbohydrate control group; KET: ketone monoester group.
